# Modeling Factors Associated with Dialysis Adequacy Using Longitudinal Data Analysis: Generalized Estimating Equation Versus Quadratic Inference Function

**DOI:** 10.34172/jrhs.2023.117

**Published:** 2023-06-30

**Authors:** Khadije Gholian, Karimollah Hajian-Tilaki, Roghayeh Akbari

**Affiliations:** ^1^Student Research Center, Research Institute, Babol University of Medical Sciences, Babol, Iran; ^2^Dept of Biostatistics and Epidemiology, School of Public Health, Babol University of Medical Sciences, Babol, Iran; ^3^Social Determinants Research Center, Research Institute, Babol University of Medical Sciences, Babol, Iran; ^4^Dept of Internal Medicine, Ayatollah Rohani Hospital, Babol University of Medical Sciences, Babol, Iran

**Keywords:** Hemodialysis, Risk factors, End-stage renal disease, Renal dialysis, Longitudinal study

## Abstract

**Background:** In hemodialysis patients, changes in dialysis adequacy (DA) are examined longitudinally. The aim of this study was to determine factors affecting DA using the generalized estimating equation (GEE) and to compare them with the quadratic inference function (QIF).

**Study Design:** A longitudinal study.

**Methods:** This longitudinal study examined the records of 153 end-stage renal disease (ESRD) patients. The longitudinal data on the DA and baseline demographic and clinical characteristics were obtained from patients’ files. The GEE1, GEE2, and QIF models were fitted with different correlation structures, and then the best correlation structure was selected using the quasi-likelihood information criterion (QIC), Akaike information criterion (AIC), and Bayes information criterion (BIC) fitting criteria.

**Results:** The majority of patients (59.5%) had unfavorable DA (KT/V<1.2). Women and patients<60 years had more favorable DA. In the GEE model, the coefficients of female gender (β=0.079, 95% confidence interval [CI]: 0.032, 0.062), age at starting dialysis (β=-0.002, 95% CI: -0.004, -0.0001), hypertension (HTN, β=-0.055, 95% CI: -0.007, -0.103), diabetes (β=-0.088,95% CI: -0.021, -0.155), dialysis duration (β=0.132, 95% CI: 0.085, 0.178), and weight (β=-0.004, 95% CI: -0.006, -0.003) demonstrated a significant relationship with DA. The three models resulted in a similar estimate of regression coefficients. The relative efficiencies of QIF versus GEE1, QIF versus GEE2, and GEE2 versus GEE1 were 1.175, 1.056, and 1.113, respectively.

**Conclusion:** DA is not optimal in most hemodialysis patients, and gender, age at the start of dialysis, HTN, diabetes, dialysis duration, and weight had a significant association with DA. The three different models yielded quite similar coefficient estimates, but the QIF model resulted more efficient than GEE1 and GEE2.

## Background

 Chronic kidney disease (CKD) is an irreversible and progressive deterioration of kidney function. The main treatment is kidney transplantation, but patients must undergo dialysis until finding a transplanted kidney. Each year, more than 60 000 people worldwide die as a result of kidney disease. The incidence of chronic kidney failure is 242 cases per million people globally and is increasing by 8% annually.^1^ The annual increase of this disease is reported to be 10%-12% in Iran.^2^ The high prevalence of this disease continued to cause high costs in the health care system worldwide.^3^ In a meta-analysis on the global burden of CKD, the highest CKD prevalence (14.44%) was found in the United States and Canada, followed by 12.10%, 11.86%, and 11.68% in Chile, Europe, and Iran, respectively, while the lowest rate of 6.76% was observed in India and Bangladesh.^4^

 Biochemical markers are routinely measured to monitor the nutritional and health status of end-stage renal disease (ESRD) patients on dialysis, which is one of the alternative treatments for ESRD patients.^5^ The dialysis adequacy (DA) index is determined based on the difference between pre- and post-dialysis urea measurements compared to pre-dialysis measurements at each dialysis session. The measurement of DA in these patients is highly important. In the case of severe inadequate dialysis, when chronic kidney failure reaches the end stage, it is impossible for the patient to continue living without replacement therapy. However, kidney transplantation is impossible in all patients.^6^ Therefore, the adequacy of hemodialysis must always be reviewed in hemodialysis patients. Various methods are employed to determine this adequacy, which is influenced by the ratio of the urea before and after dialysis. The method proposed by Daugirdas is most widely used in clinical practice.^7^ Previous reports from the Islamic Republic of Iran have shown that DA is low compared to developing countries.^8^ Several factors such as demographic and clinical characteristics may affect DA. However, none of the published studies considered the longitudinal structure of DA data and its correlation structure in their analysis.

 To monitor the health status of hemodialysis patients, the DA index and biochemical markers such as creatinine and albumin, urea, hemoglobin, hematocrit, blood lipids, and blood pressure are measured regularly. Changes in these markers during dialysis and related factors are of particular importance in predicting these changes in the nutritional assessment of patients and monitoring their health.^9^ These data are collected almost exclusively in a longitudinal setting. One of the appropriate models for the analysis of this type of data is the marginal mean model, whose best-known model is the first-order generalized estimating equation (GEE1).^10,11^ In this model, the maximum likelihood estimation (MLE) considers the correlation structure a confounding parameter. Fortunately, one of the good features of the GEE model is that the coefficient estimates are consistent and unbiased even when the chosen correlation structure is misspecified.^10-13^ The second-order GEE (GEE2) estimates the parameters based on the correlation structure using the MLE method, which can promise better estimation efficiency than GEE1. A recently developed method called the quadratic inference function (QIF), introduced by Qu et al, uses the second moments in the MLE approach.^14,15^ The latter is more of a new methodology that provides a direct method for the goodness-of-fit criteria and consistency of parameter estimates and may be more efficient, and its inferences are likely to be more robust to outliers.^14^ However, this new method has rarely been utilized in statistical analysis in medical research. The objectives of this study were to demonstrate the use of GEE and QIF in modeling marginal means for longitudinal data analysis, determine factors associated with changes in DA, and compare the relative efficiency (RE) of the three methods, GEE1, GEE2, and QIF.

## Methods

###  Design and subjects 

 This was a longitudinal study of a historical cohort in which longitudinal measurements of DA were recorded at regular intervals in each hemodialysis patient. The study sample consisted of 153 ESRD patients on dialysis between 2018 and 2019 at Shahid Beheshti Hospital in Babol, Iran. Adult patients who were over 18 years and under haemodialysis were included in the study.

###  Data and measurements

 The demographic and clinical characteristics of 153 ESRD patients were recorded from patient records using a checklist. These included age, gender, and clinical history of underlying diseases such as diabetes, hypertension (HTN), lipids, cardiovascular disease, and smoking, age at starting dialysis (year), duration of dialysis history (year), duration of each dialysis session (hour), number of dialyses per week, and weight (kg). The DA index as a dependent variable was calculated longitudinally based on the pre- and post-dialysis urea measurements and the urea clearance parameter using the Daugirdas method.^7^ These measurements were routinely recorded at the end of each month during the follow-up period. During the two-year follow-up period, these measurements were taken from each patient’s records at equal intervals every three months or eight times.

###  Statistical analysis 

 In statistical analysis, first, the package MICE was used for the multiple imputations of missing data. This package performs multiple imputations using Gibbs sampling with full conditional specification for each variable with missing elements. Then, the data normality of DA was tested using the Kolmogorov-Smirnov test, and all data were satisfied with the normality assumption. GEE1, GEE2, and QIF models were applied to estimate the regression coefficients. In the current study, the association between DA as a function of time, age, gender, age at dialysis initiation, history of cardiovascular disease, HTN, cholesterol level, smoking, diabetes, dialysis duration, dialysis history, and weight as independent variables and DA as a quantitative dependent variable was estimated, and the marginal regression model was developed as follows:


$$Y=\beta_{0}+\beta_{1}\times time+\beta_{2}\times Age+\beta_{3}\times HTN\times +\beta_{4}\times Smoking+...$$

The coefficients of the models were estimated and tested with different correlation patterns using GEE1, GEE2, and QIF models. In the current study, various correlation structures (interchangeable, autoregressive, and unstructured) were employed in the analysis of correlated data. The correlation structures must be determined before estimating the correlation parameters.^16^ In the interchangeable correlation structure, both responses of a person have the same correlation. In this case, the main diameter of the correlation matrix is equal to one, and the other elements of the correlation matrix are equal in size.^16^ In the autoregressive structure (first-order autoregressive), when the repeated observations within the individual depend on the previous observation, autoregressive correlation is applied to estimate the parameters.^15,16^ This correlation structure is mostly used because only one correlation parameter is estimated in this case. In this structure, the correlation value between two measurements of the same person equals the base correlation value multiplied by the absolute value of the time interval of these two measurements. In the unstructured method, there are fewer constraints on correlation parameters. In other words, there are different correlations for both observations within the same individual. This type of structure is used when none of the systematic structures are applicable.^16-20^ The choice of correlation structures depends on the opinion of the researcher, and the correlation structure that best reflects the relationship between correlations is not always defined clearly. For large samples, the standard error estimates of the parameters rely more on the correlation structure than on parameter estimates.^20^

 In fitting the GEE model, the Akaike information criterion is not appropriate for selecting models based on likelihood. The GEE model is based on the pseudo-likelihood; for the selection of the best performing correlation structure and comparison of the models, a statistic called the quasi-likelihood information criterion (QIC) criterion was employed, which contains the pseudo-likelihood information. The model fit is better and more appropriate when the value is lower.^20-23^ Thus, the fit of GEE1 and GEE2 was assessed with the statistical fit index QIC, QICu, and the QIF was also compared with the statistical fit index Akaike information criterion (AIC) and Bayes information criterion (BIC). In addition, the efficiency of each of these models was compared using the mean square error index of the coefficients and the estimated RE. The mice software was utilized for missing data imputation. Additionally, the library of QIF, GEE, and geepack in R version 4.0.3 software and SPSS software were used for data analysis, and statistical tests were considered at a significance level of 0.05.

## Results

###  Demographic and clinical characteristics 

**Figure 1:**
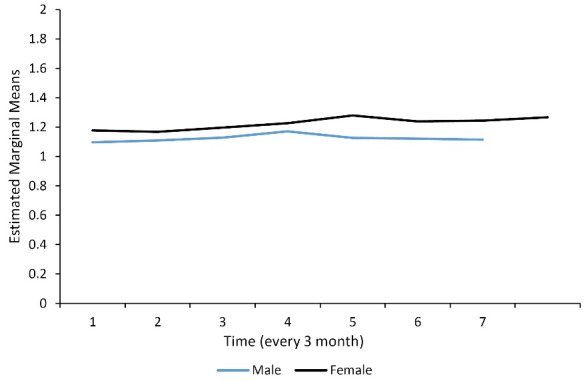
The estimated marginal means of dialysis adequacy according to gender (KT/V)

**Figure 2:**
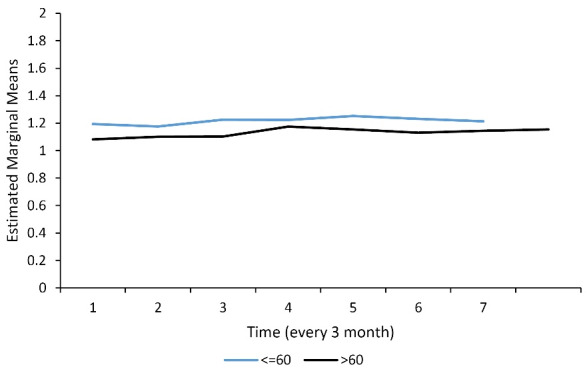
The estimated marginal means of dialysis adequacy according to age group (KT/V)

 Of the 153 ESRD patients who underwent hemodialysis, 78 (51.3%) and 74 (48.7%) cases were men and women, respectively. The missing data of DA at 8-time points of follow-up were ranged from 0% to 13%. The mean (SD) of DA and the rate of dialysis inadequacy (KT/V < 1.2) were 1.16 ± 0.24 (KT/V) and 40.5%, respectively. The DA index (KT/V) was more desirable in women than in men (Figure 1) and in the age group < 60 years compared with ≥ 65 years (Figure 2). The clinical characteristics of the study participants are presented in Table 1. HTN, cardiovascular disease, smoking, high cholesterol, and diabetes were present in 138 (90.2%), 72 (47.1%), 51 (33.3%), 73 (47.7%), and 16 (10.5%) subjects, respectively. The mean age (SD) and dialysis experience were 58.34 ± 14.19 and 4.07 ± 3.9 years, respectively.

**Table 1:** Demographic and Clinical Characteristics of ESRD Patients

**Characteristics**	**Number**	**Percent**
Gender		
Male	78	51.3
Female	74	48.7
Age group (y)		
≤ 60	76	50.3
> 60	75	49.7
History of hypertension		
No	14	9.2
Yes	138	90.7
History of cardiovascular disease		
No	81	52.9
Yes	72	47.1
Smoking history		
No	102	66.7
Yes	51	33.3
History of high cholesterol		
No	80	52.3
Yes	73	47.7
History of diabetes		
No	137	89.5
Yes	16	10.5
Dialysis adequacy (KT/V)		
≤ 1.2	91	59.5
> 1.2	62	40.5
Quantitative variables	**Mean**	**SD**
Age (year)	58.34	14.19
Dialysis adequacy (KV/T)	1.16	0.24
Age of starting dialysis (year)	54.46	15.25
History of dialysis (year)	4.07	3.90
Diastolic blood pressure (mm Hg)	81.44	6.33
Systolic blood pressure (mm Hg)	139.61	15.17
Height (cm)	164.25	6.25
Weight (kg)	68.79	14.83
Triglyceride (mg/dL)	135.25	55.71
Cholesterol (mg/dL)	131.66	27.75
Dialysis duration (h)	3.58	0.43

*Note*. ESRD: End-stage renal disease; SD: Standard deviation.

###  Factors associated with dialysis adequacy 

Table 2 provides the estimated coefficients (95% CI) of the regression parameters in the GEE1, GEE2, and QIF models. The correlation structure that produced the lowest AIC, BIC, and QIC fitting criteria was presented among various tested correlation structures. The three models produced rather similar estimates of the regression coefficients of factors associated with DA. For example, in the GEE1 model, age at starting dialysis (*P* = 0.003), HTN (*P* = 0.023), diabetes (*P* = 0.007), and weight (*P* = 0.001) had a negative significant effect, while female gender (*P* = 0.002) and duration of dialysis (*P* = 0.001) exerted a positive significant impact on DA. The coefficient of age, high cholesterol, smoking, history of cardiovascular disease, and number of dialyses per week had no significant influence on DA. In fitting GEE1 and GEE2, the QIC and QICu were lower for the unstructured correlation than for the other correlation structures. However, in fitting QIF, the best correlation structure was exchangeable with lower AIC and BIC. Thus, it was chosen as the appropriate structure.

**Table 2:** The Regression Coefficients (95% CI) of Factors Associated With Dialysis Adequacy and Fitting Criteria Using the Three Models of GEE1, GEE2, and QIF

**Parameters**	**GEE1 (Unstructured)**			**GEE2 (Unstructured)**			**QIF (Exchangeable)**		
	**β (95% CI)**	**SE**	***P *** **value**	**β (95% CI)**	**SE**	***P *** **value**	**Β (95% CI)**	**SE**	***P *** **value**
y-intercept	0.966 (0.710, 1.220)	0.131	0.001	0.966 (0.727, 1.205)	0.122	0.001	0.928 (0.694, 1.161)	0.119	0.001
Time (3 m)	0.003 (-0.002, 0.008)	0003	0.291	0.003 (-0.002, 0.008)	0.003	0.291	0.005 (-0.001, 0.010)	0.003	0.086
Gender (female vs. male)	0.079 (0.032, 0.126)	0.023	0.002	0.079 (0.029, 0.128)	0.025	0.002	0.076 (0.025, 0.125)	0.025	0.003
Age (year)	-0.001 (-0.064, 0.062)	0.032	0.976	-0.001 (-0.062, 0.061)	0.031	0.976	-0.016 (-0.078, 0.046)	0.031	0.618
Age of starting dialysis (year)	-0.002 (-0.004, -0.0001)	0.001	0.033	-0.002 (-0.004, -0.0002)	0.001	0.033	-0.002 (-0.003, 0.0003)	0.001	0.114
History of HTN (yes vs. no)	-0.055 (-0.007, -0.103)	0.025	0.023	-0.055 (-0.008, -0.102)	0.024	0.023	-0.054 (-0.003, -0.104)	0.025	0.036
History of diabetes (yes vs. no)	-0.088 (-0.021, -0.155)	0.034	0.007	-0.088 (-0.024, -0.153)	0.033	0.007	-0.070 (-0.013, -0.127)	0.029	0.015
History of high cholesterol (yes vs. no)	0.003 (-0.037, 0.042)	0.020	0.885	0.003 (-0.037, 0.043)	0.020	0.885	0.007 (-0.033, 0.046)	0.020	0.733
Smoking history (yes vs. no)	-0.011 (-0.059, 0.004)	0.025	0.655	-0.011 (-0.062, 0.039)	0.026	0.655	-0.008 (-0.058, 0.042)	0.025	0.765
History of cardiovascular diseases (yes vs. no)	-0.019 (-0.063, 0.023)	0.022	0.397	-0.019 (-0.066, 0.026)	0.024	0.397	-0.023 (-0.069, 0.022)	0.023	0.320
History of dialysis (year)	0.001 (-0.004, 0.007)	0.003	0.536	0.002 (-0.003,0.006)	0.003	0.536	0.001 (-0.003, 0.006)	0.002	0.560
Dialysis duration (hour)	0.132 (0.085, 0.178)	0.024	0.001	0.131 (0.086, 0.177)	0.023	0.001	0.151 (0.106, 0.195)	0.022	0.001
Number of dialysis per week	0.013 (-0.035, 0.062)	0.025	0.551	0.013 (-0.031, 0.058)	0.023	0.551	0.010 (-0.033, 0.053)	0.022	0.652
Weight (kg)	-0.004 (-0.006, -0.003)	0.001	0.001	-0.004 (-0.006, -0.003)	0.001	0.001	-0.005 (-0.006, -0.003)	0.001	0.001
Q	-	-	-	-	-	-	4.015	-	0.995
AIC	-	-	-	-	-	-	32.015	-	-
BIC	-	-	-	-	-	-	74.441	-	-
QIC	1274.154	-	-	1274.154	-	-	-	-	-
QICu	1236.264	-	-	1236.264	-	-	-	-	-

*Note*. CI: Confidence interval; SE: Standard error; GEE1: First-order generalized estimating equation; GEE2: Second-order generalized estimating equation; QIF: Quadratic inference function.

###  Comparison of the relative efficiency quadratic inference function vs. generalized estimating equation

 The RE of three presented models (i.e., GEE1, GEE2, and QIF) was calculated using the formula presented by Qu et al.^14^ The results revealed that the GEE2 model was more efficient than GEE1 (RE = 1.113), and the QIF model was more efficient than GEE1 and GEE2 (RE = 1.175 and RE = 1.056, respectively).

## Discussion

 The results of the GEE1 and GEE2 methods for the DA response showed that the estimates of the regression coefficients were quite similar for both methods, and there was no significant difference between the two methods, which is similar to the statistical significance of the parameter estimates. As for the RE of the coefficient estimates, the GEE2 method performed better than the GEE1 method. It is also known that the GEE1 model is used to estimate the marginal mean parameters, and the correlation parameters are not estimated in this model. This version of GEE may lead to inefficient estimates of the regression parameters because the MLE method considers correlation parameters as nuisance parameters.^23-25^ However, the GEE2 model estimates not only the marginal mean parameters but also the dependence correlation parameters. This may enhance the reliability of the estimates of regression coefficients. However, in a study by Zayeri et al,^26^ both GEE1 and GEE2 methods were applied to the continuous microleakage rate data. Further, the correlation between replicates was not significant, and the estimates of both GEE1 and GEE2 methods were almost similar.

 The results of the ongoing study suggested that the QIF method was more efficient than GEE1 and GEE2. However, the estimates of the regression coefficients were similar to GEE1 and GEE2. The results of the current study are consistent with the findings of other studies.^23,25^ In a simulation study conducted by Qu et al using Poisson data, it was found that the QIF method is more efficient than the GEE method, and when the correlation structure was correctly identified, the GEE and QIF methods had the same efficiency.^25^ In the longitudinal study by Odueyungbo et al, the comparison between GEE and QIF demonstrated that the QIF method had better RE than GEE.^23^ In most studies, the QIF method has been proposed as a method that eliminates the shortcomings of the GEE and improves the efficiency of model parameters when the correlation is not correctly determined. A few studies also discussed that the QIF method may not provide adequate results.^27^

 In the present study, clinically interesting findings indicated that the majority of patients had inadequacy of dialysis ( < 1.2 KT/V). When dialysis treatment was first used to replace the work of the kidneys, no one knew how much dialysis treatment was needed to keep patients healthy. Despite the remarkable improvement in DA in hemodialysis patients in developed countries, the majority of patients in developing countries, including Iran, still have a KT/V < 1.2. According to the 2006 NKF/DOQI criteria, the target value for KT/V is 1.4, and KT/V above 1.2 is considered an acceptable value for DA, which was used in the present study. Many factors contribute to the failure to achieve the minimum dialysis requirements, including frequent episodes of low blood pressure, poor access to health resources, incorrect nutritional status, blood flow rate, type of filtration, and also variations in sampling, and/or information errors.^27-31^

 In the current study, the quality of dialysis had a statistically significant relationship with patient gender (i.e., women were more satisfied with the adequacy of dialysis than men), which is consistent with the findings of a study by Taziki and Kashi in other dialysis centers in northern Iran.^32^ This finding may be explained by lower muscle mass, less physical activity, and diet compliance in women. The higher level of DA in women, compared with men, is also consistent with the results of other studies.^33-38^

 The findings of the ongoing study indicated that a history of HTN, diabetes, weight gain, dialysis history, and duration of dialysis were significantly related to DA. In another study on Iranian dialysis patients, a significant relationship was found between weight and DA (i.e., patients with higher weight had lower adequacy).^3^ In another study, the mean DA was low, and gender, smoking, dialysis history, number of dialysis sessions per week, and duration of dialysis also had a significant relationship with DA.^4^ In studies conducted between 1999 and 2004 in dialysis centers in the south, east, center, west, and north of Iran, 86%, 90%, 80%, 100%, and 58% of patients had a KT/V ≤ 1.2 as inadequate dialysis, respectively.^32-34^ However, in the present study, dialysis inadequacy was 41%, indicating that the level of DA in Iranian patients has improved compared to two previous decades. Nevertheless, the level of DA in the current study was lower than in developed countries. Higher levels of DA have been reported in this regard. For example, Del Pozo et al^35^ and Maduell et al in Spain reported a value of 1.3 KT/V and 0.98 KT/V, respectively.^36^ In contrast, in a recent study by Saeedi et al in Iran, the majority of patients (74.8%) had no DA; the mean DA was extremely low, and gender, dialysis history, number of dialysis sessions per week, dialysis duration, and smoking history were significantly related to the KT/V value.^37^ The difference in DA may be explained by the power of the rotation dialysis machine, duration of dialysis, the experience of the staff that operating the dialysis machine, and some patients’ characteristics.

 Moreover, in the present study, the average dialysis time was 3.58 hours per session, which was lower than the average dialysis time in Taiwan (4.53 hours) and in all of Europe (5.4 hours), but it was almost as long as the average dialysis time in the United States (3.68 hours) and Germany (3.7 hours).^38^ Although time is an influential and independent factor in the quality of dialysis, it is influenced by the number of patients, the alarm time of the dialysis machine, the efficiency of the machine, the qualification of the staff, and the rotation of the machine per minute. Increasing dialysis duration may improve the quality of dialysis to some extent. Some hemodialysis center staff are aware of this issue but may not pay attention to it due to the high number of patients on each shift and inadequate equipment and resources.

 This study had limitations. Although the study setting was a central dialysis facility covering more than half a million people in northern Iran, this study may limit the generalizability of the results. A future prospective multicenter study of ESRD patients may overcome this limitation. However, this study has several advantages in terms of longitudinal analysis to include the correlational structure of longitudinal data of DA as a quantitative dependent variable, whereas the published study almost exclusively used a cross-sectional data analysis of DA with a conventional regression model.

**Highlights**

- The rate of dialysis inadequacy (KT/V < 1.2) was 40.5%, and the dialysis adequacy (DA) index (KT/V) was more desirable in women and the age group < 60 years.
- There was a significant relationship between the DA index and female gender, hypertension, diabetes, history of dialysis, dialysis duration, and weight.
- QIF, GEE1, and GEE2 yielded similar estimates of the effect size of affecting factors on DA.
- The estimated regression coefficients of affecting factors on DA by QIF are more efficient than GEE1 and GEE2.

## Conclusion

 The results of the present study indicated that the majority of hemodialysis patients had an inadequate dialysis value (KT/V ≤ 1.2). History of HTN, diabetes, dialysis duration, and weight had a negative impact on DA. Based on the findings, GEE1, GEE2, and QIF models yielded quite similar estimates of the regression coefficients, while the QIF model led to higher efficiency in estimating the coefficients compared with GEE1 and GEE2.

## Acknowledgments

 The authors would like to thank the Research and Technology Vice-chancellor of Babol University of Medical Sciences and the esteemed staff of the Dialysis Department of Shahid Beheshti Hospital, Babol, Iran for their sincere cooperation.

## Author’s Contribution

**Conceptualization: **Karimollah Hajian-Tilaki, Khadigeh Gholian, Roghayeh Akbari.

**Data curation: **Khadigeh Gholian, Roghayeh Akbari.

**Formal analysis: **Karimollah Hajian-Tilaki, Khadigeh Gholian.

**Investigation: **Karimollah Hajian-Tilaki, Khadigeh Gholian, Roghayeh Akbari.

**Methodology: **Karimollah Hajian-Tilaki, Khadigeh Gholian.

**Project administration: **Khadigeh Gholian.

**Resource:** Karimollah Hajian-Tilaki.

**Software: **Khadigeh Golian.

**Supervision: **Karimollah Hajian-Tilaki.

**Validation: **Karimollah Hajian-Tilaki, Khadigeh Gholian.

**Visualiztion: **Karimollah Hajian-Tilaki, Khadigeh Gholian, Roghayeh Akbari.

**Writing–original draft: **Khadigeh Gholian.

**Writing–review & editing: **Karimollah Hajian-Tilaki, Khadigeh Gholian, Roghayeh Akbari.

## Competing Interests

 The authors declare that they have no conflict of interests.

## Ethical Approval

 This study was approved by the Ethics Committee of Babol University of Medical Sciences (Ethics code: IR.MUBABOL.REC.1400.110). Research involving human participants or human data was performed in accordance with the Declaration of Helsinki. All participants of the study were informed about the study, and they gave written informed consent to be included in the study.

## Funding

 Not applicable.
